# Effect of ECAP Route Type on the Microstructural Evolution, Crystallographic Texture, Electrochemical Behavior and Mechanical Properties of ZK30 Biodegradable Magnesium Alloy

**DOI:** 10.3390/ma15176088

**Published:** 2022-09-02

**Authors:** Abdulrahman I. Alateyah, Majed O. Alawad, Talal A. Aljohani, Waleed H. El-Garaihy

**Affiliations:** 1 Department of Mechanical Engineering, College of Engineering, Qassim University, Unaizah 56452, Saudi Arabia; 2 Materials Science Research Institute, King Abdulaziz City for Science and Technology (KACST), Riyadh 12354, Saudi Arabia; 3 Mechanical Engineering Department, Faculty of Engineering, Suez Canal University, Ismailia 41522, Egypt

**Keywords:** equal channel angular pressing, route type, ultrafine-grained structure, corrosion behavior, potentiodynamic polarization, electrochemical impedance spectroscopy

## Abstract

In this study, billets of the ZK30 (Mg-3Zn-0.6 Zr-0.4 Mn, wt%) alloy were Equal Channel Angle Pressing (ECAP) processed for up to four passes of routes Bc (with rotating the sample 90° in the same direction between the subsequent passes), A (without sample rotation), and C (with sample rotating 180°) after each pass at a temperature of 250 °C and a ram speed of 10 mm/min using a die with an internal channel angle of 90°. The microstructural evolution and the crystallographic texture were investigated using a Scanning Electron Microscope (SEM) equipped with the Electron Back-Scatter Diffraction (EBSD) technique. Corrosion measurements were conducted in ringer lactate which is a simulated body fluid. The Vickers microhardness test and tensile tests were conducted for the alloy before and after processing. The as-annealed billets exhibited a bimodal structure as fine grains (more than 3.39 µm) coexisted with almost-equiaxed coarse grains (less than 76.73 µm); the average grain size was 26.69 µm. Further processing until four passes resulted in enhanced grain refinement and full Dynamic Recrystallization (DRX). ECAP processing through 4-Bc, 4-A, and 4-C exhibited significant reductions in grain size until they reached 1.94 µm, 2.89 µm, and 2.25 µm, respectively. Four-pass processing also resulted in the transformation of low-angle grain boundaries into high-angle grain boundaries. The previous conclusion was drawn from observing the simultaneous decrease in the fraction of low-angle grain boundaries and an increase in the fraction of high-angle grain boundaries. The pole figures revealed that 4-Bc, 4-A, and 4-C reduced the maximum texture intensity of the as-annealed billets. The potentiodynamic polarization findings revealed that route Bc is the most effective route in improving the corrosion rate, whereas the Electrochemical Impedance Spectroscopy (EIS) revealed that routes A and Bc improved the corrosion resistance with nearly identical values. Finally, 4-Bc resulted in the highest increase in Vickers hardness, yield stress, and ultimate tensile strength with values of 80.8%, 19.3%, and 44.5%, alongside a 31% improvement in ductility, all compared to the AA condition.

## 1. Introduction

Magnesium alloys (Mg) have been known to scientists to be excellent potential materials for the manufacturing of bioresorbable orthopaedic implants. Scientists expect that Mg alloys may replace currently used materials like titanium and cobalt-chromium alloys on their merits of being more biocompatible and thus less likely to cause the body to have allergic or sensitization reactions [1,2,3,4,5,6]. In actuality, Mg alloys’ bioresorbability in the human body is sufficient enough to omit the need for a removal surgery as it degrades naturally, which is an advantage over the currently used materials [7,8]. Mg alloys are also extremely biocompatible with the human body as both share similar mechanical properties. For example, these alloys are characteristically the lightest amongst other metals and possess densities (1.75 up to 1.85 g/cm^3^) overlapping with those of human cortical bones (1.75–2 g/cm^3^) [7,9,10]. Finally, the modulus of elasticity of Mg alloys (41–45 GPa) is more similar to that of bones than any of the commonly-used materials. This provides another advantage to Mg alloys over their commonly-used counterparts as they would cause fewer stresses at the bone/implant interface [9,11].

There are four main categories of Mg-based bioresorbable materials, according to Aljihmani et al.: pure Mg, alloys containing aluminum, alloys containing rare-earth elements, and aluminum-less alloys [12]. Among the last category, aluminum-free Mg-based alloys, special light is shed on Mg-Zn alloys. Mg-Zn alloys prove to be viable contenders as materials for bioresorbable implants. There are many advantages to using these alloys. Firstly, Zn is already present in the human body and enters many biological processes such as wound healing and the formation of bones [9,13]. Secondly, a Zn wt% of 3 in Mg leads to the formation of a heterogenous second phase, MgZn_2_, which improves the alloy’s tensile and corrosive properties [14,15,16]. Thirdly, Zn acts as a cathode in some environments, although it is not very effective [16]. Finally, the addition of Zn further strengthens the alloy through solid solution strengthening, grain refinement, as well as through other mechanisms like increasing the alloy’s age hardening response [1,5,17]. In addition, Zn assists in resisting the corrosive effect of impurities like nickel and iron and hence, improves the corrosion behavior of Mg alloys [18]. ZKxx alloys, or Mg-Zn-Zr, where Z is zinc and K is Zirconium, prove themselves to be great biocompatible substrates for implants and other medical applications due to their strength and low cytotoxicity propelled by their lack of aluminum, contrary to most Mg-based bioresorbable alloys [1,3,5,14]. Compared to a lot of medically used alloys, ZKxx alloys display superior strength and corrosion resistance after being subjected to grain refining processes. This is owed to Zr which facilitates the improvement of these properties [19]. 

Implants support the body while the bones heal, so at the minimum, the implant must outlast the healing period [1]. Although Mg alloys’ natural tendency to degrade in the human body is a sought-after property in bone implants, the rate at which the alloy degrades is a risk factor. Mg alloys have a problematic tendency to fail prematurely from corrosion which causes their mechanical properties to wane and become insufficient to serve their role [5]. One possible solution to avoid the aforementioned problem is solid-solution strengthening Mg by alloying it with Zn and Zr, which significantly boosts Mg’s corrosion properties. Further improvement of corrosion properties can be realized through the grain refinement of the alloy, which has been proven to boost metals’ mechanical properties as well [5,20,21,22,23]. Severe Plastic Deformation (SPD) is an umbrella term comprised of various metal forming techniques that are known for their ability to produce Ultrafine-Grained (UFG) metals. SPD techniques increase metallic materials’ strength by creating nano-sized secondary phases that are scattered homogenously throughout the structure [5,20,21,24,25,26]. Valiev et al. were the first to foresee the practical applications of applying SPD techniques to biomedical implants [27]. The most prominent SPD techniques for UFG creation are Equal Channel Angular Pressing (ECAP) [28,29,30,31,32,33], High Pressure Torsion (HPT) [34,35,36], twist extrusion [37], accumulative rolling bonding [38], and Multi-Channel Spiral Twist Extrusion (MCSTE) [39,40,41,42]. Amongst the aforementioned techniques, ECAP is well-known as the most dependable and effective of them at creating UFG or Nano-Structured (NS) metallic materials, which is reflected in the produced material’s mechanical and corrosion properties [25,28,43,44,45].

**Figure 1:**
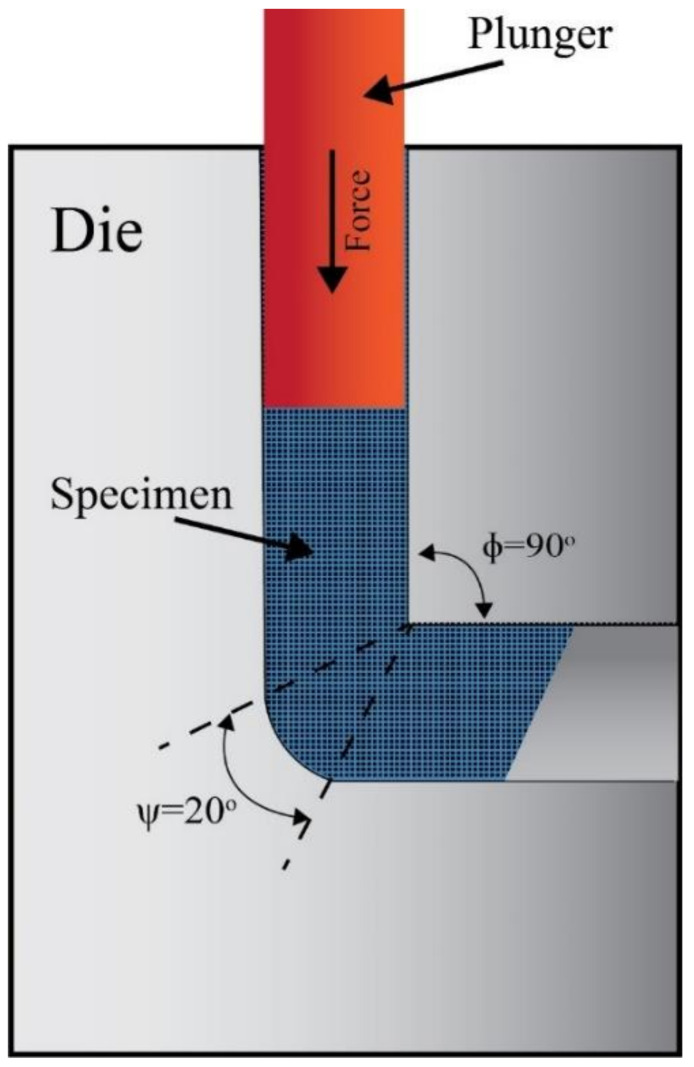
The schematic of the ECAP die.

ECAP occurs in a metallic die consisting of two channels through which the sample is pressed with a force to induce an intense shearing strain in the sample. The two channels have equal cross-sections, and the controllable die parameters are the inner angle Φ at which the channels intersect and the angle Ψ of the outer arc of curvature joining the two channels, as shown in Figure 1 [4,21,28,31]. Different deformation routes can be applied to a sample across the multiple die-passes applied to them to create different strain profiles. Different route configurations are identified by how the sample is re-oriented around its longitudinal axis after each pass [46]. The ECAP routes most commonly applied are route A, route BA, route Bc, and route C. In route A, no form of rotation is applied to the sample between each pass. In route BA, the sample is subjected to a 90° rotation, and the direction of the next rotation is flipped after each pass (a clockwise 90° rotation followed by a counterclockwise 90° the pass after). Route Bc indicates that the sample has been continuously rotated 90° without flipping the direction of rotation after each pass. Finally, in route C, the sample is rotated 180° after each pass [31,47]. Amongst the mentioned routes, route Bc provides the most grain refinement [30], whereas route A produces the highest density of elongated grains [48]. However, applying route C an odd number of times results in elongated grains while applying it an even number of times results in matching strain profiles across the sample and produces equiaxed grains [49]. Different route types induce varying effects on the crystallographic texture and its intensity, which in turn affects the sample’s properties [50].

There have been various studies on the effects that applying SPD techniques such as ECAP has on the corrosion properties of Mg alloys, specifically on how the UFG structure that ECAP produces enhances corrosion resistance. A study on ZKxx alloys in a chloride solution has concluded that ECAP processing led to grain refinement and a more homogenous distribution of the added alloying metals, which ultimately resulted in boosted corrosion properties in the alloy [14]. Orlov et al. [14] correlated the enhanced corrosion rate of ZK60 and the grain refinement and the redistribution of Zn and Zr after ECAP processing through one-pass using an ECAP die with a special design. A number of studies have been concerned with the behavior of bioresorbable Mg alloys in simulated body fluids. Examples of investigated alloys are Mg-Zn-Zr [5], AZ31 [21,51], ZK60 [26], and ZN20 [52]. El-Garaihy et al. [5] reported that ECAP processing of the Mg-Zn-Zr alloy through 4-Bc experienced a significant decrease in the grain size of 92.7% which improved the corrosion rate by 94% compared to the as-annealed (AA) counterpart. Alateyah et al. [21] reported the improvement of the corrosion rate of AZ31 by 78.3% coupled with improving the Vickers microhardness by 132% after processing through 2-Bc using an ECAP die with an internal channel angle of 90°. Mostaed et al. [26] reported the improvement in the corrosion rate of ZK60 coupled with a significant reduction in the mechanical properties and a significant improvement in the ductility of 100% after processing through up to eight passes of route Bc. Peron et al. [51] studied the effect of ECAP processing on the stress corrosion cracking of AZ31. Gao et al. [52] reported the improvement in the corrosion resistance of the Mg-Zn-Nd alloy coupled with grain refinement, texture weakening, and enhancing the ductility by 40% after ECAP processing through four passes.

A review and analysis of previous literature written on the effects of the ECAP deformation route type on microstructural evolution and crystallographic texture led the authors to conclude that it had not been described and investigated sufficiently. Moreover, the authors believe that the relationships between ECAP processing and the corrosion behavior of the biodegradable ZK30 (Mg-3Zn-0.6 Zr wt%) alloy have not been adequately mapped. As such, the work herein investigates the aforementioned relationships through the study of the effects that different ECAP deformation routes have on the microstructural evolution, crystallographic texture, second-phase redistribution, corrosion behavior, and mechanical properties of the ZK30 alloy. The overarching goal of this work is to advance the current knowledge of the electrochemical properties of UFG Mg alloys as well as provide insight on how to adapt Mg alloys for biomedical usage. The investigated samples were processed via ECAP for either one pass (1-P) or four passes of routes A, Bc, and C. The microstructure evolution and the crystallographic texture evolution were studied through Scanning Electron Microscopy (SEM) and the Electron Back-Scatter Diffraction (EBSD) technique as well. Electrochemical tests were conducted in a simulated body fluid of ringer lactate to mimic the environment in which medical implants would be utilized. In terms of experiments, Open Circuit Potential (OCP), potentiodynamic polarization, and Electrochemical Impedance Ppectroscopy (EIS) were conducted. All experiments were also conducted on as-received samples after their annealing so that they could be used as references.

## 2. Materials and Methods

In preparation for the experiments, a commercial ZK30 (Mg-3Zn-0.6 Zr-0.4 Mn, wt%) alloy was sectioned into cylindrical billets 60 mm in length and 10 mm in radius. Prior to ECAP processing, the Mg alloy billets were annealed for 16 h at a temperature of 430 °C. The billets were then processed at a temperature of 250 °C and a ram speed of 10 mm/min through an ECAP die of Φ = 90° and Ψ = 20° as shown in Figure 1. Four ECAP routes were investigated: one pass (1-P); four passes of route Bc (4-Bc), A(4-A), and C (4-C); and one billet was left in the AA condition to serve as a reference.

The microstructural evolution was studied using a longitudinal cross-section from the centre of the billets. The samples were sectioned accordingly and cold-mounted in conductive epoxy. The samples were grinded incrementally on a grinding wheel spinning at 150 rpm using silicon-carbide sandpaper. Sandpaper of increasing grits was used (600/800/1000/1200), and before switching to higher grit sandpaper, each specimen was rinsed out with water. Then the samples were polished using diamond suspensions of particle sizes 3 μm then 1 μm mixed with yellow DP-lubricant. All samples were to have scratch-free surfaces as seen using a microscope. To that end, a final polishing step was conducted; a 0.05-micron colloidal silica formula was used to provide the final polish. Polishing was conducted on a rotating wheel paten spinning at 150 rpm, and between polishing rounds, the specimen was ultrasonically cleaned in ethanol for 10 min. Samples were then etched in a solution of 100 mL ethanol, 5 mL acetic acid (95%), 6 g picric acid, and 10 mL water for 50 s. Finally, to remove the top amorphous layer, the samples were flat ion milled for 30 min using a flat ion milling system. The milling parameters were a grazing angle of 5°, a specimen rotational speed of 0.425 s^−1^, and beam energy of 2 keV.

The microstructure evolution following ECAP was investigated using a SU-70 SEM capable of performing EBSD which was used to characterize the microstructural and crystallographic texture evolution as well. The samples investigated by the SEM and EBSD were sectioned from the central longitudinal plane of the ECAPed billets parallel to the pressing direction. The axes of the reference system coincide with the extrusion ECAP direction (ED). The SEM operated at 15 kV and 1.5 nA, and the EBSD data were collected in 100 nm increments from the top surface ED plan using HKL Flamenco Channel 5 software (Hitachi, Ltd., Tokyo, Japan). Inverse Pole Figure (IPF) maps were created using the post-processing features of the same software. Elemental analysis techniques were used to investigate the elemental composition of the alloy, namely Energy-Dispersive X-ray Spectroscopy (EDS) and X-ray Fluorescence (XRF). For XRF analysis, the ECAP billet was sectioned to have a 20 mm diameter and 5 mm thickness. The crystal structure was characterized using X-ray diffraction (XRD) and a JEOL JDX-8030 X-ray diffractometer. The diffractometer operated using a voltage of 40 kV, a current of 30 mA, and Cu-Kα radiation at a scan rate of 2 degrees/min.

Multiple corrosion tests were conducted on the samples to understand the evolution of the ZK30 alloy’s corrosion properties after ECAP processing. All tests were conducted, and all data were recorded through an SP-200 Potentiostat (Bio-Logic, Orlando, FL, USA). Three chief types of tests were conducted. Firstly, the Open Circuit Potential of the samples was assessed. Secondly, cyclic and linear (potentiodynamic) polarization techniques were applied with a potential range of ±250 mV against OCP and a scan rate of 0.166 mVs^−1^—to ensure steady-state behavior. Finally, EIS experiments were conducted at OCP through the application of sinusoidal voltages with a potential window of ±10 mV and with frequencies ranging between 100–1000 kHz. The setup in which the experiments took place was a 3-electrode flat corrosion cell in which the counter electrode was a piece of platinum mesh, the reference electrode was a Saturated Calomel Electrode (SCE), and the samples themselves were the working electrodes after they were prepared for the experiment, and room-temperature ringer lactate was the corroding agent. The sample preparation process consisted of sectioning the samples into 20 × 30 mm rectangles, grinding them incrementally with 800, 1200, and 4000 grit silicon-carbide sandpaper, and finally cleaning the samples with acetone and rinsing them with deionized water. Additionally, a Haber-Luggin capillary was used to establish an accurate sensing point for the potential-sensing tip of the reference electrode.

The evolution of the ZK30 alloy’s mechanical properties after ECAP processing was analyzed using Vickers microhardness tests (Hv) and tensile tests. The hardness tests were conducted on longitudinal (LS) and transverse (TS) sections of the ECAPed samples. Each test was performed starting from the section’s peripheries and from a minimum of four equispaced indentations up until the center of the sample to provide a representative average of the sample’s HV value. The applied load used was 0.5 kg applied for 15 s. Tensile tests were conducted at room temperature on samples machined to the E8M/ASTM standard using 100 kN universal testing machines. Three specimens selected from the billet’s center were tested at a strain rate of 10^−3^ s^−1^ per processing route to ensure representative results.

## 3. Results and Discussion

### 3.1. Microstructure Evolution

**Figure 2:**
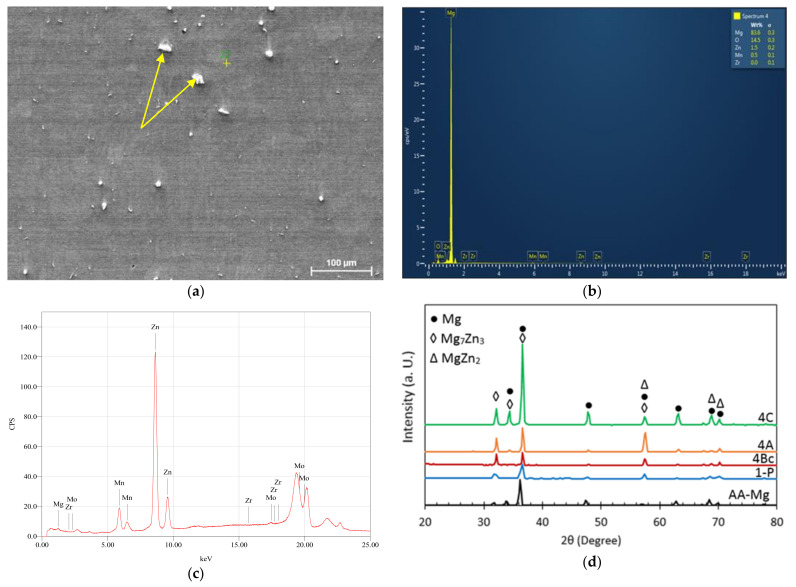
(**a**) SEM micrographs, (**b**) EDS analysis, (**c**) XRF analysis of the AA ZK30 billets, and (**d**) XRD pattern diffractions of the ZK30 alloy before and after ECAP processing; arrows point at second phases.

Figure 2 consists of an SEM micrograph (Figure 2a) of the AA billets of ZK30 coupled with the billet’s EDS analysis (Figure 2b), its XRF analysis (Figure 2c), and the XRD patterns (Figure 2d) of the AA as well as the processed billets (through 1-P, 4-Bc, 4-A, and 4-C). Figure 2a clearly depicts the secondary phases which accumulate at the grain boundaries and the triple conjunctions of the α-matrix grains. This is in line with what Orlov et al. have previously reported [14]. Furthermore, the EDS and XRF analyses confirmed the existence of Mg, Zn, Zr, and Mn elements in the AA billet through the displayed element peaks in Figure 2b,c, respectively. In addition to the α-Mg phase, the XRD analysis authenticated the existence of very weak secondary-phase peaks of MgZn_2_, Mg_7_Zn_3_, and Zn_2_Zr_3_ secondary phases, as shown in Figure 2d. The weakness in intensity can be ascribed to the annealing process. The peaks’ intensities increased after ECAP processing through 1-P which confirms that ECAP processing leads to the precipitation of secondary phases. Further processing through four passes resulted in a notable increase in the secondary phases’ peak intensities as shown in Figure 2d. Similar findings were reported in [5,14,21].

**Figure 3:**
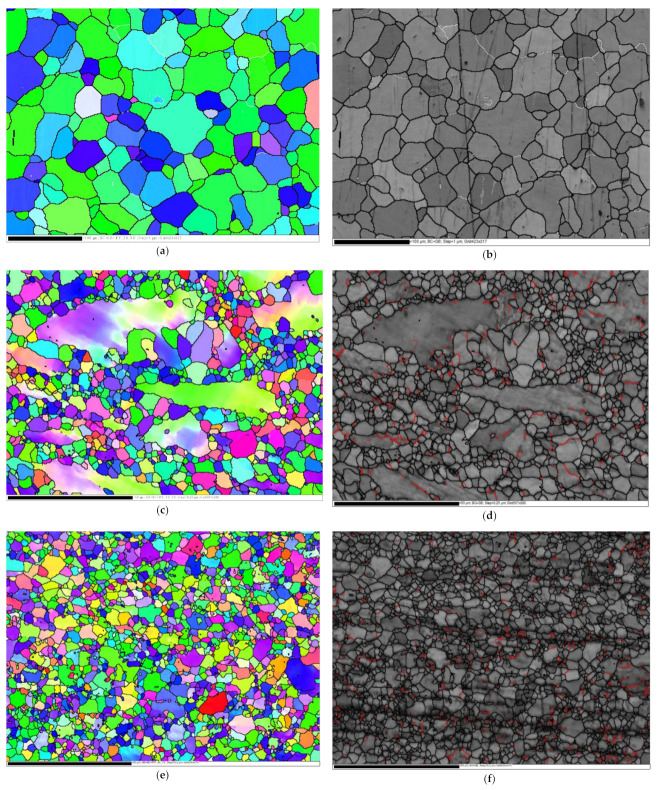

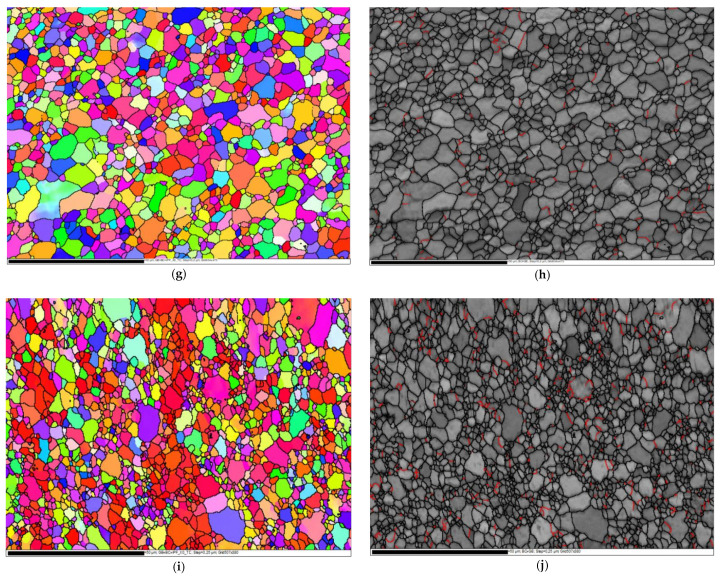
IPF colouring maps relative to ND and their corresponding BC maps with HAGBs in black lines and LAGBs in white lines (AA) and red lines (1-P, 4-Bc, 4-A, and 4-C), superimposed for the AA ZK30 billets (**a**,**b**) and ECAPed billets through (**c**,**d**) 1-P, (**e**,**f**) 4-Bc, (**g**,**h**) 4-A, and (**i**,**j**) 4-C.

**Figure 4:**
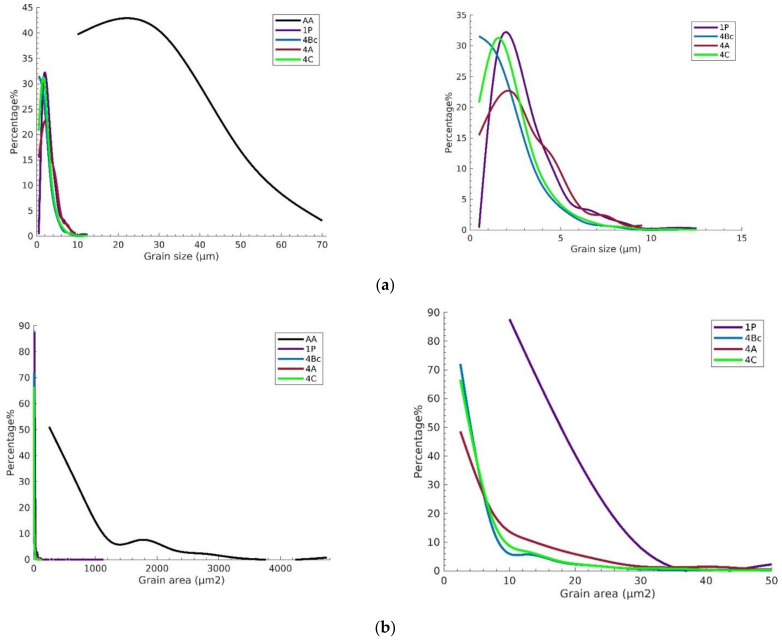

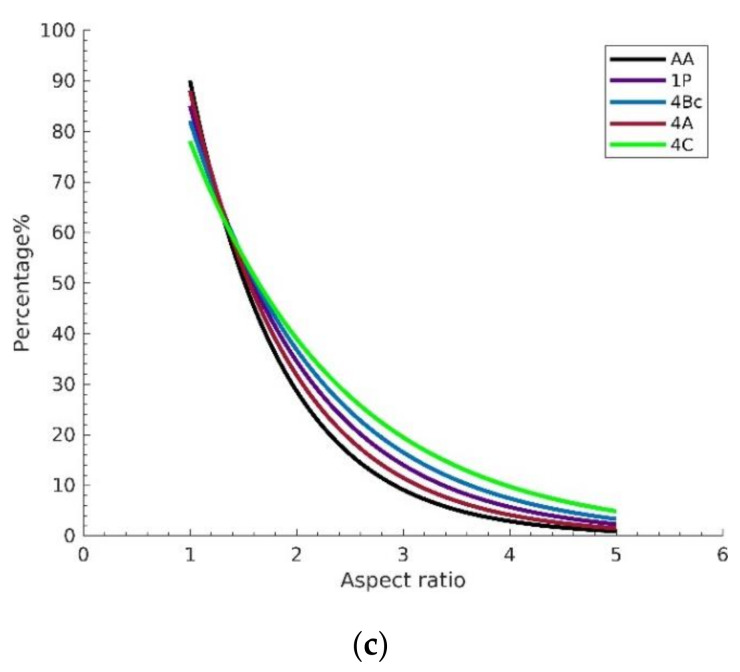
Relative frequency of (**a**) grain size, (**b**) grain area distribution, and (**c**) grain AR of all ZK30 samples.

**Figure 5:**
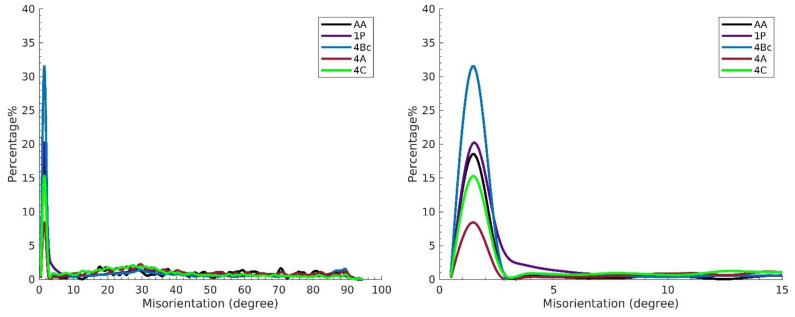
Misorientation angle distribution histograms obtained from the EBSD data for ZK 30 alloy before and after ECAP processing.

The Inverse Pole Figure (IPF) colouring maps and their band contrast (BC) maps are shown for the AA billets as well as the billets processed under each of the four routes in Figure 3. High Angle Grain Boundaries (HAGBs) are taken to be of misorientation >15°, while the misorientation angle between 3° and 15°, is a Low Angle Grain Boundary. HAGBs are coloured black while LAGBs are coloured white in the AA condition maps and red in the 1-P, 4-Bc, 4-A, and 4-C billets maps, relative to the ED as shown in Figure 3. In addition, Table 1 shows the minimum, maximum, and average grain size of the AA and the ECAPed ZK30 billets. Comparisons between grain size distribution, grain area distributions, and grain aspect ratios (AR) of the ZK30 alloy are presented in Figure 4. The LAGBs and HAGBs distributions of the ZK30 alloy before and after ECAP processing are also shown in Figure 5.

After annealing, the structure of the ZK30 billets was revealed to be bimodal: fine grains (more than 3.39 µm) were found alongside almost equiaxed coarse grains (less than 76.73 µm). The average grain size was 26.69 µm, and the average grain area was 729 µm^2^ (Figure 3a). Furthermore, it was clear that the grains were wholly recrystallized. The grain boundaries (GB) map revealed a very low number of LAGBs as shown in Figure 3b. In addition, more than 90% of the AA grains experienced a grain AR of almost 1 as shown in Figure 4c. The AA structure was dominated by the (010) (green) and (120) (blue) orientations as shown in Figure 3a. ECAP processing via 1-P resulted in severely elongated grains coexisting with a UFG structure, which indicates the occurrence of partial recrystallization as shown in Figure 3c. Similar to the AA condition, the 1P route’s structure was also dominated by the (010) and (120) orientations. As listed in Table 1, a significant reduction in the grain size was achieved as the grain size ranged from 1.13 µm to 38.1 µm with an average grain size of 3.24 µm. The previous findings indicate that after 1-P processing, the grains were refined by 87.86% compared to their AA counterpart. Furthermore, the grains’ average area was reduced by 98% compared to the AA condition (Figure 4b). On the other hand, the grains’ average AR increased by 202% compared to the AA counterpart.

In terms of the distribution of the LAGBs (3° < LAGBs < 15°), the GB maps between the AA and the 1-P samples showed a significant increase in the LAGBs’ densities (Figure 3d compared to Figure 3b). The LAGBs’ and HAGBs’ densities increased by 225% and 88% after 1-P when compared to that of the AA sample (Figure 4b compared to Figure 4a). In addition, it was clearly noted that 1-P processing led the coarse (AA) grains to split into smaller and ultra-fine grains (Figure 3c) which led to an increase in the HAGBs density. On the other hand, the UFG structure obtained after ECAP processing is linked to the increased fraction of LAGBs in the 1-P billets (Figure 3d). This finding agreed with Shana et al. [53] who proposed that ECAP processing resulted in the generation and multiplication of dislocations which then entangled with each other to form LAGBs. Thus, the LAGB density increased significantly in the early passes of ECAP.

Accumulation of the imposed shear strain up to four passes caused further grain refinement and full Dynamic Recrystallization (DRX) of the grains. Samples processed through 4-Bc exhibited a higher density of equiaxed ultra-fine grains, as shown in Figure 3e. Grain sizes ranged from 0.23 µm to 11.76 µm with an average grain size of 1.94 µm which indicates that the grains’ average size was reduced by 92.7%, compared to the AA counterpart. The 4-A processed sample experienced coarser grains and a more homogenous grain size distribution, compared to the 4-Bc condition. Grain sizes ranged from 0.23 µm to 14.53 µm with an average grain size of 2.89 µm (Table 1) which indicates that the average grain size was reduced by 89% compared to the AA condition. Processing via 4-C resulted in a fairly inhomogeneous grain size distribution. UFGs were observed in addition to some coarse grains as seen in Figure 3i. Route 4-C resulted in grain sizes ranging from 0.28 µm to 12.73 µm and an average grain size of 2.25 µm (Table 1), which was a reduction of 91.6% in the grain size compared to the sample’s AA counterpart. Accordingly, route 4-Bc has caused the most grain refinement and reduced the average grain size by 32.9% and 13.8% compared to 4-A and 4-C conditions, respectively (Figure 3 and Figure 4a). In addition, from Figure 4a, it can be seen that 4-Bc had the highest fraction of fine grains (<1.5 µm), whereas 4-C had the highest fraction of grain sizes, from 1.5 µm to 2.8 µm, and 4-A had the highest fraction of coarse grains (>2.8 µm). On the other hand, 4-Bc, 4-A, and 4-C exhibited average grain areas of 4.838, 9.475, and 5.975 µm^2^, respectively, which means that 4-Bc, 4-A, and 4-C resulted in a reduction in the average grain area of 99.3%, 98.7%, and 99.1%, respectively, compared to the AA coarse grains. Routes 4-Bc and 4-C had nearly identical grain area distribution curves whereas 4-A showed larger grain areas on average, as shown in Figure 4b. From the aforementioned findings, it was clear that route Bc achieved more refinement compared to the other routes. This can be attributed to the nature of the shear strain present in the Bc route which operates parallel to the extrusion and transverse directions (ED and TD). When processing via multiple passes, the shear planes first intersect at 120° and when the billet is rotated 90° between passes, the shear planes operated in both the y-plane and z-plane, and superior grain refinement was achieved [47]. Furthermore, during consecutive passes of route C, the shear directions were inverted parallel to each other so that the grains were deformed repetitively during ECAP resulting in the generation and accumulation of more dislocations, which results in an enhanced DRX process compared to route A [54]. In terms of the grains’ AR, 4-A revealed the highest fraction of semi-equiaxed grains (AR < 1.8), whereas 4-C revealed the highest fraction of elongated grains (1.8 < AR < 5). It can be noted that 4-Bc, 4-A, and 4-C exhibited an average grain AR of 1.485, 1.343, and 1.55, respectively. Thus, contrary to popular belief, route A is the most effective route in producing equiaxed grains whereas route C is the most effective route in producing elongated grains within the four-pass limit the ZK30 alloy was subjected to.

Concerning the LAGBs’ densities, increasing the strain up to four passes resulted in a decrease in the LAGBs’ densities compared to the 1-P condition due to dynamic recovery [5]. Processing through 4-Bc, 4-A, and 4-C caused a reduction in the LAGBs’ densities by 25.4%, 39%, and 6.78%, respectively, compared to the 1-P condition (Figure 5). On the other hand, processing through three of the four-pass routes increased the HAGBs’ densities by 4.4%, 6.77%, and 1% for the ZK30 billets processed through 4-Bc, 4-A, and 4-C, respectively, compared to the 1-P counterpart. The increase in the HAGBs’ densities after performing multiple passes can be attributed to the transformation of the LAGBs into HAGBs after the recrystallization process. This proposition is substantiated by the decrease in the LAGBs’ densities by increasing the number of passes. From the aforementioned findings, it can be noted that the 4-A route caused a higher density of LAGBs to transform into HAGBs, which resulted in a more stable structure and a better strengthening effect caused by the HAGBs blocking the dislocation movement. Route 4-C exhibited the lowest amount of LAGBs transforming into HAGBs. Thus, the samples processed through the previous route showed less strength due to the lower hindrance provided by the HAGBs against the dislocation motion. Dumitru et al. [55] explained the increase in the strength of the ZK60 alloy after ECAP processing to be caused by the accumulation and rearrangement of dislocations resulting in the formation of subgrains and fine equiaxed grains. In addition, they confirmed the increase in the LAGBs’ densities in the first pass of ECAP and their decrease in the subsequent passes. Figueiredo et al. [56] proposed a new model to describe the microstructural evolution of Mg alloys during ECAP. They suggested that the grains evolve during the early passes into a bimodal or multimodal grain distribution. Further processing passes lead to more refinement and the achievement of a homogenous UFG structure.

Many earlier studies have investigated the effects of the ECAP route type on microstructural evolution. Sankuru et al. [47] have processed pure Mg through four passes of different routes. They reported that four passes were sufficient to achieve a homogenous microstructure and to reach a steady-state grain size in all routes. Furthermore, they found that route Bc was the most effective route in grain refinement. Vaughan et al. [57] processed the Mg-ZKQX6000 alloy through hybrid routes and they reported that hybrid-route processing resulted in significant refinement in both α-grains and the precipitates at high-density shear regions. Gzyl et al. [58] subjected the AZ31B Mg alloy to multiple passes of I-ECAP through routes A, Bc, and C at different processing temperatures. They reported that the Mg billets processed through route A showed less tendency to fracture than the other routes. Tong et al. [54] processed the Mg-Zn-Ca alloy through four passes of routes A, Bc, and C. They reported that route Bc was the most effective route in grain refinement, route A was the least effective, and route C resulted in more elongated grains. Accordingly, these findings agree with the findings of this study. In addition, they recorded that the fraction of LAGBs was reduced the most and was transformed into HAGBs by the usage of route Bc, which indicates a faster DRX process compared to routes A and C. Suh et al. [59] reported that routes A, C, and D did not refine AZ31 further than 9–10 µm with an AA average grain size of 14 µm and the three routes resulted in an almost equiaxed structure. In addition, Zhou et al. [60] explained the grain refinement in the polycrystalline materials during SPD by the generation of new grain boundaries by increasing the imposed strain which leads to increasing the misorientation to accommodate the deformation. Furthermore, the Geometrically Necessary Dislocations (GNDs) produced a part of the total dislocations with a misorientation greater than 15°, thus building up misorientations between the adjacent grains.

### 3.2. Crystallographic Texture

**Figure 6:**
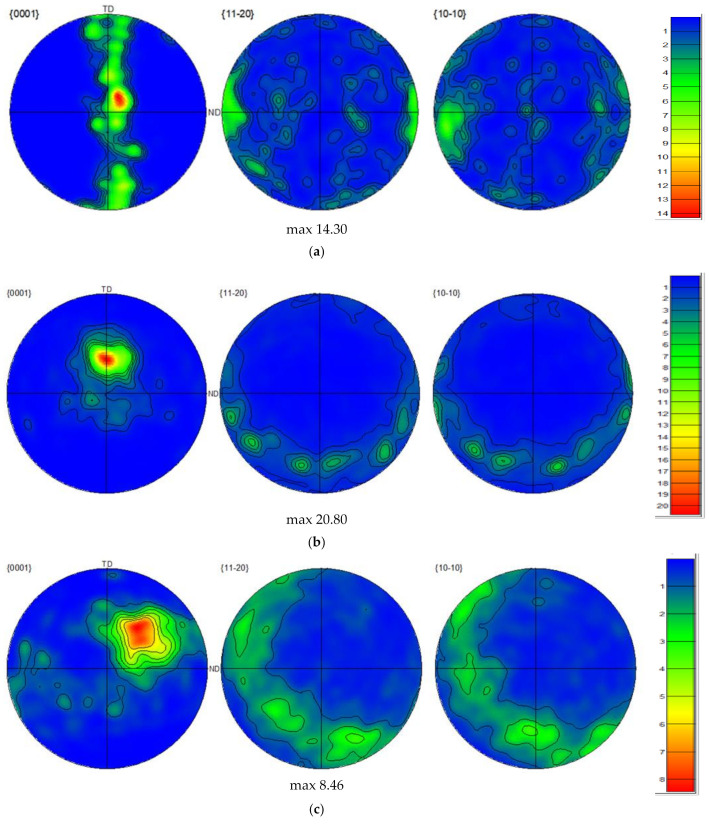

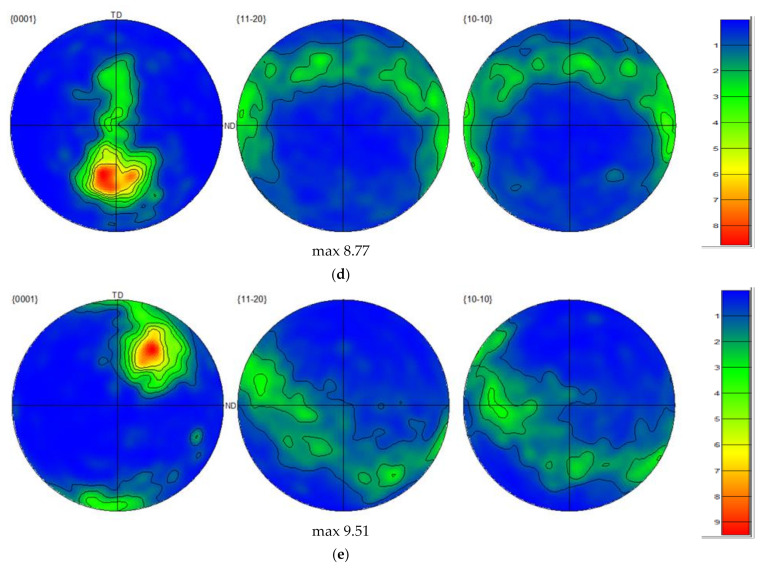
{0001}, {11-20}, and {10-10} pole figures showing the crystallographic texture of (**a**) AA, (**b**) 1-P, (**c**) 4-Bc, (**d**) 4-A, and (**e**) (4-C).

The pole figures (PF) of the AA and ECAPed ZK30 billets’ {0001}, {11-20}, and {10-10} planes are displayed in Figure 6. As shown in Figure 6a, the {0001} (basal) planes are aligned parallel to the Transversal Direction (TD) with a maximum texture intensity of 14.3 times random. On the other hand, it was observed that the poles of the {11-20} and {10-10} planes were aligned parallel to the ED. Processing through ECAP via 1-P resulted in very strong basal plane texture components with a maximum texture intensity of 20.8 times random as shown in Figure 6b. The {0001} basal planes of the 1-P condition were oriented parallel to the TD. Rotating the ECAPed billet around its central longitudinal axis during processing via route Bc led to an altered crystallographic texture as the {0001} basal planes were rotated approximately 45° relative to the TD, as shown in Figure 6c. In addition, from Figure 6c it was clear that 4-Bc processing resulted in a reduction in the maximum texture intensity to 8.46 times random. The reduction in the texture intensity after processing through 4-Bc can be attributed to the limited number of ZK30 slip systems during shear deformation [61]. Suh et al. [59] reported that the route Bc applied to the AZ31 alloy resulted in a slight increase in the texture component intensity compared to the other routes, whereas the texture component was becoming weaker in the rolling direction with additional passes, and the new texture component was developed visibly along the TD. The processing of Mg alloy billets repetitively up to four passes without any rotation between the subsequent passes (route A) resulted in the {0001} basal planes becoming oriented parallel to the TD with a similar maximum texture intensity as its 4-Bc counterpart, as shown in Figure 6d (8.77 times random). Suh et al. [59] found that route A increased the Mg alloy’s basal planes’ propensity for parallel alignment with the rolling direction and reinforced the new texture component which was formed after earlier passes. ECAP processing through 4-C—rotating the ZK30 billets 180° about their longitudinal axis between passes—led to the tilting of the {0001} basal planes approximately 45° away from the TD coupled with an increase in the maximum texture intensity to 9.51 times random as shown in Figure 6e. In addition, they reported that processing through route C resulted in a notable reduction in the texture intensity after every even pass instead of a recovery of the initial formed texture. This can be attributed to the nature of route C which applies shearing stress on the same plane but in opposite directions every two passes. The basal planes’ rotation can be attributed to the shearing stresses parallel to the basal planes during ECAP processing [62]. The rotation of most basal poles close to 45° from ED and TD was confirmed by XRD [63]. The crystallographic texture findings were in agreement with [64] for the AZ31B Mg alloy, [55] for Mg–Zn–Ca, [59] for the AZ31 alloy, and [65] for pure Mg.

### 3.3. Electrochemical Measurements

**Figure 7:**
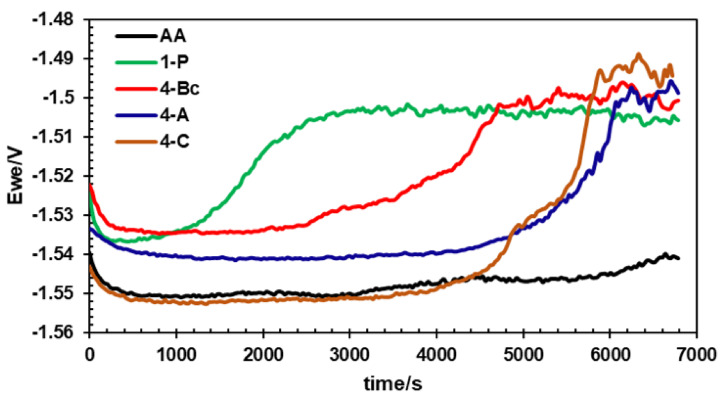
OCP curves of ZK30 alloy before and after ECAP processing.

Figure 7 shows the OCP of the AA and the ECAP-processed billets of the ZK30 magnesium alloy. From Figure 7, it is clear that the AA billets experienced a slight decrease in the corrosion potential before stabilizing at −1.55 V. Similar to the AA billet at first, the ECAPed billets displayed a slight decrease in corrosion potential, then remained at a constant potential value for a period of time, and finally, dramatically increased before coming to reach a constant value (Figure 7). For the 1-P condition, it took the sample approximately 2400 s to reach a constant potential value of −1.505 V. On the other hand, it took the 4-Bc, 4-A, and 4-C samples approximately 4600, 6000, and 5800 s to reach a constant value of −1.5, −1.5, and −1.44 V, respectively. The dramatic increase in the corrosion potential of the ECAPed billet is a clear indication of more noble behaviour compared to its AA counterpart. This can be attributed to the generation of a more stable and protective oxide layer [5]. Similar findings were reported by Mostaed et al. [24]. The notable reduction in the corrosion potential at the beginning of the OCP which was followed by a dramatic increase in the potential after a sufficient time was also reported by Yang et al. [32]. They attributed the drop in the potential to a failure in the air oxide layer until it built another passive oxide layer which resulted in the increase in corrosion potential. It is worth mentioning here that four passes of any route revealed more noble potential compared to 1-P. On the other hand, the 4-C condition revealed the noblest potential of all the samples, as shown in Figure 7.

**Figure 8:**
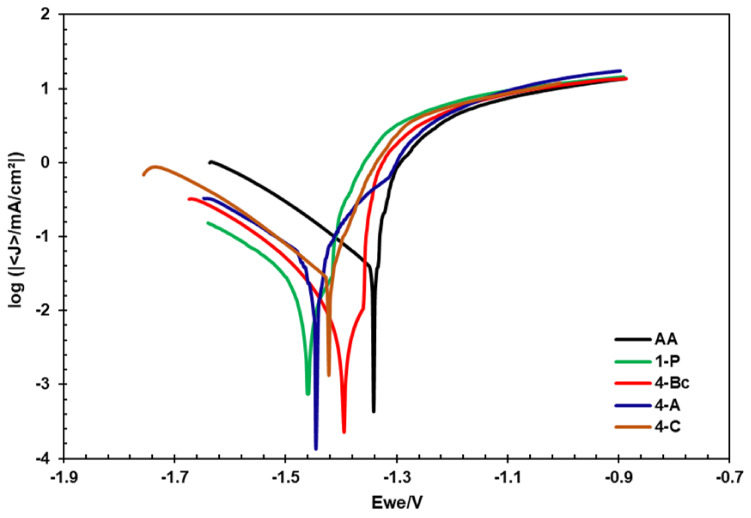
Linear potentiodynamic polarization curves of the ZK30 alloy before and after ECAP processing.

Figure 8 displays the potentiodynamic polarization curves (Tafel plots) of all the sample conditions. The corrosion current density (Icorr), corrosion potential (Ecorr), Tafel’s anodic and cathodic constants (βa and βc), and the corrosion rate in mils penetration per year (mpy) were calculated from Tafel plots and tabulated in Table 2. From Table 2 and Figure 8 it is clear that processing through 1-P caused a significant decrease in Icorr (about 87%) compared to the AA counterpart, which indicated a noble shift in the Icorr compared to the AA condition. Icorr offers a dependable method to calculate corrosion resistance. The 1-P route processing led to a significant decrease in the corrosion rate from 154.7 mpy to 20.2 mpy (about 87%) compared to the coarse-grained billets (AA), as tabulated in Table 2. Further straining to 4-Bc caused a more noble shift of the Icorr, recording a 54.8% decrease compared to its 1-P counterpart. Accordingly, the 4-Bc condition experienced a significant decrease of 94% in the ZK30 corrosion rate compared to the coarse-grained AA billets. In addition, despite showing an improved Icorr and corrosion rate compared to their AA counterpart, both 4-A and 4-C displayed lower Icorr and corrosion rate values compared to the 4-Bc condition (Figure 8). Compared to their AA counterparts, the 4-A billets revealed a significant improvement of 87.7% in the corrosion rate (Table 2). On the other hand, the 4-A sample revealed a 108.9% increase in the corrosion rate compared to its 4-Bc counterpart. Similar to the 4-A billets, the 4-C billets experienced a notable corrosion rate improvement of 83% compared to the AA, while on the other hand, they showed a notable increase of 185.7% in corrosion rate, compared to their 4-Bc counterparts.

**Figure 9:**
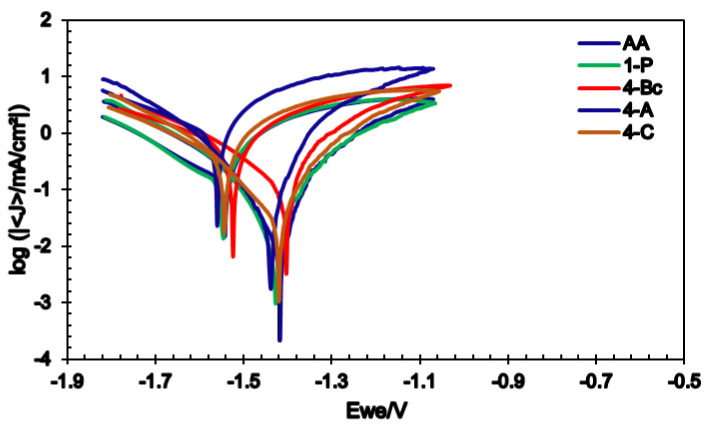
Cyclic potentiodynamic polarization of ZK30 alloy before and after ECAP processing.

The cyclic potentiodynamic polarization (CPD) plots of the AA and ECAPed ZK30 billets, processed through different conditions, are shown in Figure 9. From Figure 9, it is clear that both the AA and ECAPed billets exhibited a complete hysteresis loop which confirmed the ability of ZK30 billets to re-passivate and revealed its corrosion protection potential [66]. Furthermore, it was clear that all the processed billets exhibited nobler potential in the forward scan Ecorr than in the back scan. This confirms the Mg alloy’s re-passivation potential and hence corrosion resistance, which agrees with Lui et al. [67]. It is worth mentioning that ECAP processing caused an Icorr decrease in the passive region, which confirms the role ECAP processing has in the formation of the protective layer with a higher thickness compared to the AA. This agrees with [5,21,68,69]. Finally, it was reported that the better the grain refinement, the more the improvement of the Mg alloy’s re-passivation ability [69].

**Figure 10:**
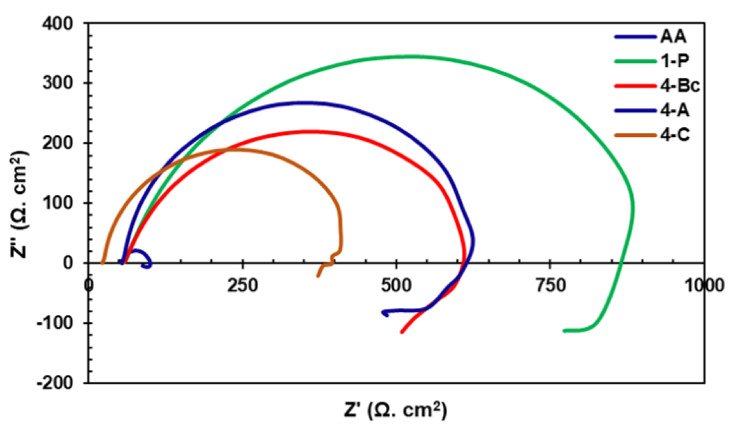
Nyquist plots of ZK30 alloy before and after ECAP processing.

**Figure 11:**
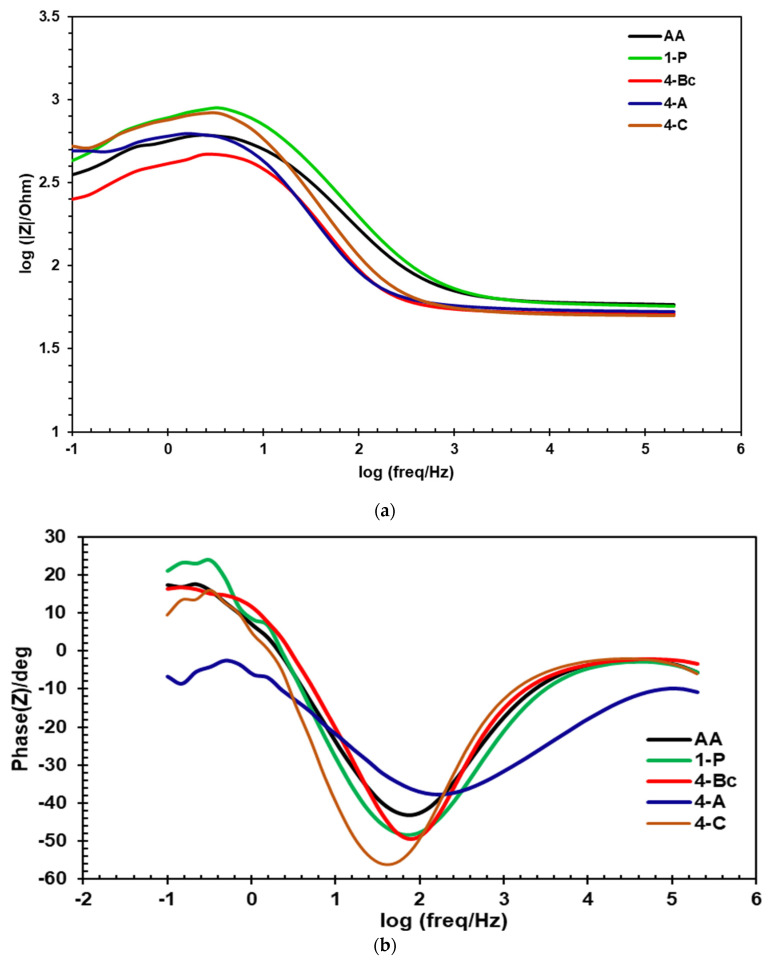
(**a**) Bode plot, (**b**) Bode plot phase angle of ZK30 alloy before and after ECAP processing.

**Figure 12:**
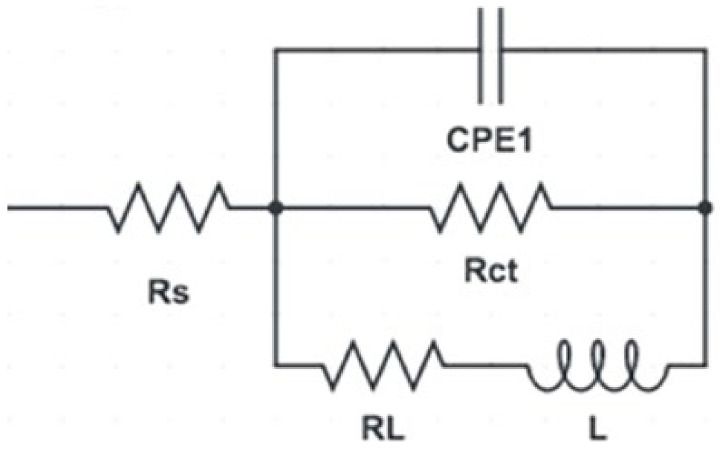
Equivalent circuit used to fit (EIS) data.

EIS was carried out to further investigate the effect of the ECAP processing route on the corrosion resistance of ZK30 billets. Both Nyquist plots and Bode magnitude plots are presented in Figure 10 and Figure 11a, respectively. In addition, the Bode phase plot of ZK30 billets before and after ECAP processing is shown in Figure 11b. Furthermore, the equivalent circuit used to fit the EIS data is shown in Figure 12. On top of that, the electrical parameters of the EIS equivalent circuit are shown in Table 3. The solution resistance is donated by Rs, the double-layer charge transfer resistance at the ZK30 alloy/solution interface is donated by Rct, the pitting resistance is donated by R_L_, the inductance is denoted by L, and CPE corresponds to the double-layer capacitance.

As shown in the ZK30 Nyquist plots for (Figure 10), the AA and the ECAPed billets exhibited capacitive semicircles. In addition, the AA plot revealed a diminutive capacitive arc compared to its processed counterparts. This indicates a significant improvement in corrosion resistance after ECAP processing. Accordingly, the EIS findings confirmed the effectiveness of the grain refinement occurring from ECAP processing in improving the corrosion resistance of the ZK30 alloy. ECAP processing through 1-P resulted in a significant increase in the capacitive arc, as shown in Figure 10. On the other hand, further processing through four passes of any of the routes displayed a smaller capacitive arc compared to the 1-P counterpart. Naik et al. [70] reported the reduction in corrosion resistance in subsequent passes of the AZ80 alloy. They justified this reduction as a result of the increase in dislocation density caused by the accumulation of strain from multiple passes of ECAP processing. The same could be attributed to the decrease in the corrosion resistance of the Mg alloy. Gopi et al. [71] reported that the dislocation build-up—resulting from multiple-pass ECAP processing—stored an abundant quantity of internal energy as grain boundary energy, which accelerated pitting corrosion. Furthermore, 4-Bc and 4-A conditions had seemingly identical capacitive arcs, whereas the 4-C route showed a much smaller capacitive arc.

The Bode plots of the ZK30 billets shown in Figure 11a revealed that the 1-P condition exhibited the highest corrosion resistance at almost all frequencies, whereas the 4-C condition revealed a higher corrosion resistance at low and medium frequencies, compared to 4-Bc and 4-A. Furthermore, 4-A exhibited higher corrosion resistance at low frequencies compared to its 4-Bc counterpart, whereas they exhibited almost identical corrosion resistance at both medium and higher frequencies, as shown in Figure 11a. From Figure 11b, it is clear that 4-Bc had the highest phase angles amongst all samples at lower frequencies, whereas 4-A had the highest phase angles at intermediate frequencies, and both 4-Bc and 4-C had similar phase angles, which were the highest among other samples at higher frequencies. Accordingly, the Bode plot shown in Figure 11a reasserted the improved corrosion resistance of the ECAPed ZK30 billets compared to their AA counterparts.

The result from 1-P processing was an increase in both the Rct and R_L_ by 1131.3% and 151.5%, respectively, compared to the AA condition (Table 3). Strain accumulation from 4-Bc, 4-A, and 4-C processing caused a notable reduction in both Rct and RL compared to their 1-P counterpart. Analyzing the EIS data of the 4-Bc condition revealed 726.4% and 143.5% increases in the Rct and RL, respectively, compared to the AA condition, whereas the 4-A condition revealed nearly identical increases of 731.2% and 144.9% in Rct and RL, respectively, compared to the AA condition as tabulated in Table 3. On the other hand, the 4-C condition exhibited the lowest improvements in Rct and RL with increases of 554.76% and 76.35%, respectively, compared to the AA counterpart. Accordingly, EIS findings agreed with both the microstructural evolution (Figure 3) and potentiodynamic polarization findings (Figure 8).

The improved corrosion rate of Mg alloys can be attributed to the significant reduction in grain size after multiple-pass processing through ECAP, as well as the generation of a protective oxide layer [24]. The higher refinement in grain size, especially for the 4-Bc condition, resulted in lower corrosion rates (9.1 mpy) compared to 4-A and 4-C, which were verified via the potentiodynamic polarization findings and EIS findings in this study. In addition, the higher the deformation of the Mg alloy, the greater the thickness of the protective oxide layer, resulting in more corrosion resistance and a lower corrosion rate, as confirmed in earlier studies [5,21,68]. Significant grain refinement led to a thicker protective passive oxide layer and enhanced its coherency; hence, grain refinement leads to better protection against pitting corrosion in Mg alloys [14,69]. Similar findings were reported by Gurao et al. [72] for commercially pure titanium processed through three passes of routes Bc, A, and C. They reported that 3-Bc exhibited the lowest Icorr and corrosion rate compared to the coarse-grained Ti billets followed by 3-A, whereas 3-C exhibited the highest Icorr and corrosion rate among all the three processing routes. In addition, they reported that the corrosion rate is directly linked to the material’s grain size, the orientation of the texture, and grain boundary character. In addition, they concluded that applying processing routes that result in an off-basal texture and produce UFG can lead to higher corrosion resistance. In contrast, Rifai et al. [73] found that increasing the number of passes applied to the Fe-20%Cr alloy resulted in increased Ecorr and Icorr. They also reported that route C had more stable Tafel polarization curves than routes A and Bc, and they attributed this to the high fraction of HAGBs and grain boundary misorientations present. Gebril et al. [74] reported that amongst the routes they tested, route Bc was the most effective in improving the corrosion resistance of the A356 Alloy. They attributed that to the higher refinement of the structure produced by route Bc compared to route A.

**Figure 13:**
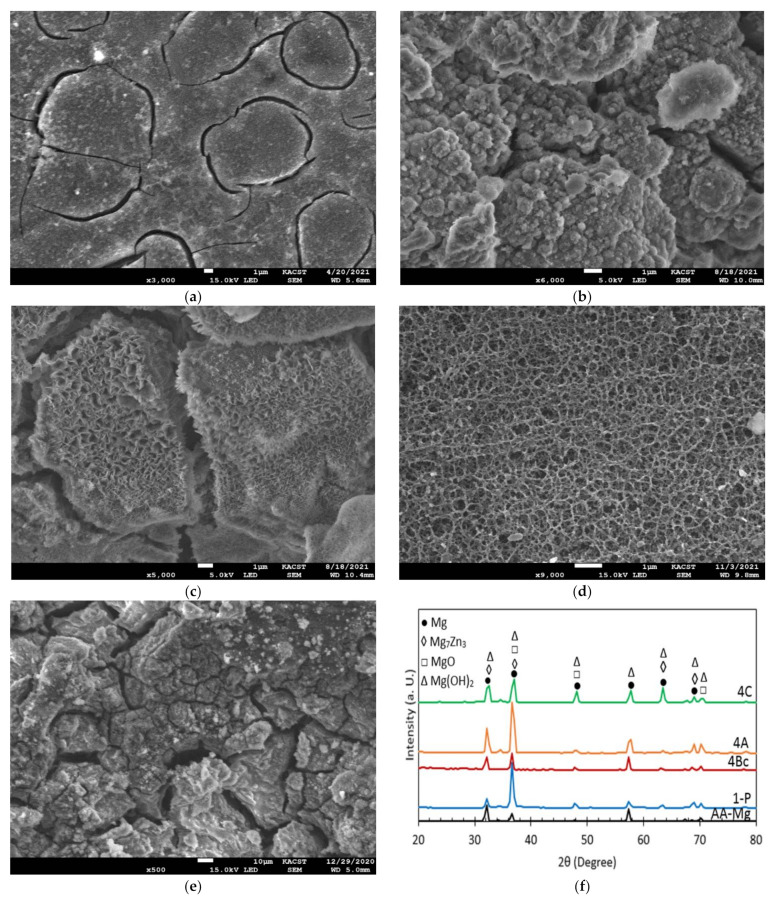
(**a**–**e**) SEM micrographs of the ZK30 alloy after corrosion testing. AA (**a**), ECAP processed through routes 1-P (**a**), 4-Bc (**c**), 4-A (**d**), 4-C (**e**), and (**f**) X-ray diffraction patterns of ZK30 alloy after corrosion.

In addition to producing a UFG structure that affects the corrosion behaviour of Mg alloys when processed by ECAP for multiple passes, SPD processing also results in a high density of dislocation sites [75,76]. Accordingly, these areas of high dislocation densities improve the corrosion resistance of the Mg alloy as it forms protective hydroxide and oxide layers (Mg(OH)_2_ and MgO) [5,21,24,77]. Furthermore, multiple-pass ECAP processing increases the thickness, stability, and coherency of the protective layer as well [49,77]. Figure 13 shows SEM micrographs of the AA and ECAPed ZK30 billets after corrosion tests. In addition, the XRD pattern of the Mg alloy after corrosion is shown in Figure 9f. Highly corroded areas were evident in the AA condition whereas hydroxide and oxide protective layers manifested in ECAP processed samples. The accumulation of the imposed strain from processing up to four passes resulted in a thicker protective layer, compared to the 1-P condition (Figure 13). It was also noted that the 4-A condition displayed the densest, most coherent, and uniformly distributed protective layer among all the processing conditions. Accordingly, the SEM micrographs are in good agreement with the EIS findings. Finally, the XRD pattern shown in Figure 13f confirmed the presence of Mg(OH)_2_ and MgO which agrees with an earlier study [71]. Notably, the ECAPed billets’ oxide protective layers displayed higher intensity peaks compared to their AA counterparts.

The improvement in the corrosion resistance resulting from an increased number of processing passes was reported in earlier studies. Tang et al. [77] attributed this improvement in the corrosion resistance of AZ80 to the obtained UFG structure and to the grain refinement of the secondary phases which were uniformly distributed throughout the grain boundaries. Cubides et al. [78] reported an increase in the corrosion resistance of AZ91 with the increase in the number of ECAP passes. However, Sadawy et al. [79] attributed this enhancement in corrosion resistance after ECAP processing to the UFG structure, and to a reduction in the number of segregated impurities at the grain boundaries. Alateyah et al. [21] reported that ECAP processing resulted not only in the refinement of the α-Mg grains but the secondary phases as well, which were distributed homogeneously along the grain boundaries and inside the α-Mg grains. In addition, they found that increasing the number of ECAP passes improved the corrosion resistance by improving the coherency of the oxide protective layer.

### 3.4. Mechanical Properties

The Vickers microhardness values (HV) of the ZK30 alloy were measured along the billet’s transverse section. Table 4 shows the HV-values of the AA as well as the ECAPed billets processed through 1-P, 4-Bc, 4-A, and 4-C. The HV values recorded from the AA billets were similar along the billet’s transverse section, with an average of 52 HV. ECAP processing through 1-P caused a significant increase of 63.5% in the HV-value compared to its AA counterpart. Accumulation of strain from increasing the number of processing passes up to four caused a further increase in the HV-values without exception. The 4-Bc condition experienced the highest increase in HV-value of 80.8% compared to its AA counterpart. The 4-A condition had a 75% increase in HV value when put in comparison with the AA condition. Finally, the 4-C condition displayed a 77% increase in the HV-value compared to the AA sample, as shown in Table 4. The significant increase in the HV-value after 1-P can be attributed to the significant grain refinement as well as the increased texture intensity of 1-P compared to the AA, which agrees with the IPF (Figure 3) and pole figures findings (Figure 6). Four-pass processing caused additional grain refinement; hence, higher HV-values were recorded. Table 1 shows that 4-Bc is the most effective route type in grain refinement. Thus, it follows that 4-Bc would have higher hardness than its 4-A and 4-C counterparts. Accordingly, both grain boundary and strain hardening strengthening were the chief strengthening mechanisms imparted by ECAP processing to the ZK30 alloy, which agrees with earlier studies [28,70]. Similar findings were reported by Sankuru et al. [47] who reported that 4-Bc was the most effective route in improving the hardness values of pure Mg; however, no significant difference in hardness values was recorded among all ECAP processing routes. Illgen et al. [49] reported that route Dc (rotating the sample 90° in the same direction along the ND between subsequent passes) was the most effective.

**Figure 14:**
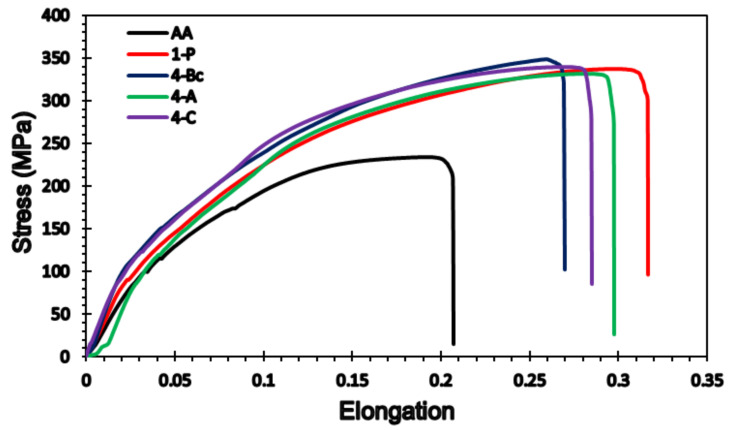
Stress–strain curves of ZK30 billets before and after ECAP processing.

In addition to the microhardness measurements, the tensile properties of ZK30 billets in the AA and ECAP processed conditions were assessed at room temperature. The stress-strain curves of the ZK30 alloy are presented in Figure 14. Furthermore, the yield stress (σy), ultimate tensile strength (σu), and elongation at fracture (εf) values are tabulated in Table 4. From Figure 14, it is clear that the ECAP processing of the Mg alloy did not significantly alter σy. It resulted in a notable increase in σu coupled with a significant improvement in εf. ECAP processing through 1-P caused a 12% increase in σy compared to its AA counterpart. On the other hand, strain accumulation from processing via 4-Bc, 4-A, and 4-C caused 19.3%, 16.25, and 18.75% increases in σy, respectively, when put in comparison with the AA condition.

Despite achieving a significant reduction in grain size through ECAP processing as shown in Figure 3, the improvement in σy does not agree with the expected rise in strength following such refinement. This indicates that the crystallographic texture following ECAP processing has countered the strengthening caused by grain size refinement, which agrees with earlier studies [28,47,62]. In addition, it was reported by Lie et al. [80] that the activation of non-basal slip systems severely impacts the σy of the Mg alloys. On the other hand, ECAP processing through 1-P resulted in a significant increase of 39.5% in σu, compared to the AA condition. Furthermore, increasing the number of processing passes up to 4-Bc, 4-A, and 4-C increased the σu by 44.5%, 38.6%, and 42%, respectively, compared to the AA counterpart. Accordingly, the σu findings agree with the microstructural evolution (Figure 3). Finally, ECAP processing through 1-P caused a significant increase of 53.4% in εf, compared to its AA counterpart (Figure 14). Straining the samples further through 4-Bc, 4-A, and 4-C showed notable reductions of 14.5%, 6%, and 9.8% in εf when compared with the 1-P condition. The increase in the εf of Mg after ECAP processing has been reported in an earlier study [47].

Based on the aforementioned findings of the mechanical properties of the ZK30 alloy, it is clear that the ECAP-induced grain refinement plays a vital role in strengthening the Mg alloy. This is in line with the Hall–Petch relationship and is confirmed by the increased σu and HV-values after ECAP processing. The grain refinement after ECAP processing can be attributed to the increase in dislocation density [47]. It was reported earlier that ECAP processing through multiple passes resulted in the gradual transformation of the LAGBs into HAGBs, which is intertwined with the formation of UFG [28]. In the same context, the formation of a UFG structure increased the grain boundary area and hence generated an effective barrier against the dislocation motion. This increases both the alloy’s strength and hardness which lies in agreement with [81]. The presence and uniform distribution of the second phase particles at the grain boundaries and at triple conjunction points after ECAP processing also play important roles in the alloy’s strengthening [53]. On the other hand, the notable improvement in the material’s ductility (Figure 14) is attributed to the bimodal grain structure, as shown in Figure 3 and presented in Table 1. Accordingly, the fine grains strengthened the ZK30 alloy, whereas the large grains carried higher deformation by providing strain hardening, as reported in previous studies [62,82,83]. The increase in the HAGBs’ fractions with the number of processing passes led to more sliding between the grain boundaries, hence the improved ZK30 ductility, which agrees with [55]. A similar finding was reported by Sankuru et al. [47]. They reported an increase in the σy, σu, and Ɛf of pure Mg after ECAP processing. They also reported that route Bc is the most effective route in improving σy, whereas route BA is the most effective in improving σu. On the other hand, route C was the most effective route in improving the ductility of pure Mg. They also reported that, after the first pass, a significant change in the tensile properties of the ECAPed billets was achieved; however, subsequent passes only led to minor changes in the mechanical properties, regardless of the processing route. They outlined that the starting basal slip clutches the great majority of the deformation, while the subsequent passes involved the activation of non-basal slip systems and so required a lower amount of deformation energy [47]. Alateyah et al. [84] generated a comprehensive statistical analysis of the effect of ECAP parameters on the grain size and mechanical properties of pure Mg. They reported that 2-Bc using an ECAP die with a channel angle of 120° was the optimum processing condition for increasing σu, whereas 4-C using an ECAP die with a channel angle of 120° was the optimum processing condition for improving the ductility.

## 4. Conclusions

The effects of ECAP-processing route types on the microstructural evolution, crystallographic texture, corrosion behaviour, and mechanical properties of the ZK30 Mg alloy have been investigated in this study. Up to four passes of different ECAP routes (Bc, A, and C) have been applied on the ZK30 Mg alloy at 250 °C. The following conclusions were drawn:

1. ECAP processing through 4-Bc, 4-A, and 4-C resulted in significant grain refinements of 92.7%, 89%, and 91.6%, respectively, compared to the AA counterparts;
2. Route A is the most effective route in transforming LAGBs into HAGBs;
3. ECAP processing through 4-A reduced the fraction of LAGBs by 39% and was accompanied by an increase of 6.77% in the fraction of HAGBs, compared to the 1-P counterpart;
4. The AA showed a maximum texture intensity of 14, which increased to 21 times random after 1-P. Processing through further passes led to a decrease in the maximum texture intensity;
5. The 4-Bc condition reduced the ZK30 alloy’s corrosion rate by 94%, compared to the AA billets;
6. Processing through 4-A increased the values of Rct and RL by 731.2% and 144.9%, respectively, compared to the AA condition;
7. The 4-Bc sample experienced the highest increase in Vickers hardness at 80.8%, compared to the AA condition;
8. Compared to the AA counterpart, the 4-Bc condition showed the highest increase in the yield stress and ultimate tensile strength of 19.3% and 44.5%, respectively, as well as an improvement of 31% in the ductility of the Mg alloy.

## Figures and Tables

**Table 1:** Grain size data of the AA and ECAPed ZK30 billets. All units are in µm.

	AA	1-P	4-Bc	4-A	4-C
Min	3.39	1.13	0.23	0.23	0.28
Max	76.73	38.10	11.76	14.53	12.73
Average	26.69	3.24	1.94	2.89	2.25
St. Deviation	14.74	2.42	1.54	1.92	1.60

**Table 2:** Electrochemical parameters obtained from potentiodynamic polarization curves of the AA condition and ECAP-processed ZK30 Mg alloys.

Condition	βa (mV·dec^−1^)	−βc (mV·dec^−1^)	Ecorr (V/SCE)	Icorr (µAcm^−2^)	Corrosion Rate (mpy)
AA	109.0	251.5	−1.511	168.68	154.7
1-P	64.2	185.5	−1.550	22.01	20.2
4-Bc	42.1	126.5	−1.541	9.94	9.1
4-A	56.6	122.5	−1.46	20.7	19.01
4-C	51.9	172.2	−1.42	28.4	26.0

**Table 3:** Electrical parameters obtained from fitting the EIS data of the AA condition and ECAP-processed ZK30 Mg alloys.

Condition	Rs (Ω·cm^2^)	CPE (F)	Rct (Ω·cm^2^)	R_L_ (Ω·cm^2^)	L (H·cm^−2^)
AA	58.3	81.0 × 10^−6^	69.2	191.6	43.1
1-P	58.5	26.9 × 10^−6^	852.1	482	490.2
4-Bc	59.0	36.8 × 10^−6^	571.9	466.6	424.9
4-A	64.2	20 × 10^−6^	575.2	469.3	632.7
4-C	41.1	53.9 × 10^−6^	453.1	371.8	337.9

**Table 4:** Hardness values and the tensile properties of ZK30 billets before and after ECAP processing.

Condition	Hardness HV	Yield Stress (MPa)	Ultimate Strength (MPa)	Elongation (EL%)
AA	52 ± 1	80 ± 1	238 ± 1	20.6 ± 0.5
1-P	85 ± 0.5	92 ± 2	332 ± 2	31.6 ± 0.5
4-Bc	94 ± 1	95.5 ± 1	344 ± 2	27 ± 0.1
4-A	91 ± 1	93 ± 0.5	330 ± 1	29.7 ± 0.5
4-C	92 ± 0.5	95 ± 1	338 ± 1	28.5 ± 0.5

## Data Availability

All the raw data supporting the conclusion of this paper were provided by the authors.
